# Bioinspired Design Rules from Highly Mineralized Natural Composites for Two-Dimensional Composite Design

**DOI:** 10.3390/biomimetics8060500

**Published:** 2023-10-20

**Authors:** Anamika Prasad, Vikas Varshney, Dhriti Nepal, Geoffrey J. Frank

**Affiliations:** 1 Department of Biomedical Engineering, Florida International University, Miami, FL 33174, USA; 2 Department of Mechanical and Materials Engineering, Florida International University, Miami, FL 33174, USA; 3 Materials and Manufacturing Directorate, Air Force Research Laboratory, Wright-Patterson Air Force Base, Dayton, OH 45433, USA; vikas.varshney.2@us.af.mil (V.V.); dhriti.nepal.1@us.af.mil (D.N.); geoffrey.frank@udri.udayton.edu (G.J.F.); 4 University of Dayton Research Institute, Dayton, OH 45469, USA

**Keywords:** MXene, 2D materials, bioinspired design, nacre, biomimetic mineralization, multiscale modeling

## Abstract

Discoveries of two-dimensional (2D) materials, exemplified by the recent entry of MXene, have ushered in a new era of multifunctional materials for applications from electronics to biomedical sensors due to their superior combination of mechanical, chemical, and electrical properties. MXene, for example, can be designed for specialized applications using a plethora of element combinations and surface termination layers, making them attractive for highly optimized multifunctional composites. Although multiple critical engineering applications demand that such composites balance specialized functions with mechanical demands, the current knowledge of the mechanical performance and optimized traits necessary for such composite design is severely limited. In response to this pressing need, this paper critically reviews structure–function connections for highly mineralized 2D natural composites, such as nacre and exoskeletal of windowpane oysters, to extract fundamental bioinspired design principles that provide pathways for multifunctional 2D-based engineered systems. This paper highlights key bioinspired design features, including controlling flake geometry, enhancing interface interlocks, and utilizing polymer interphases, to address the limitations of the current design. Challenges in processing, such as flake size control and incorporating interlocking mechanisms of tablet stitching and nanotube forest, are discussed along with alternative potential solutions, such as roughened interfaces and surface waviness. Finally, this paper discusses future perspectives and opportunities, including bridging the gap between theory and practice with multiscale modeling and machine learning design approaches. Overall, this review underscores the potential of bioinspired design for engineered 2D composites while acknowledging the complexities involved and providing valuable insights for researchers and engineers in this rapidly evolving field.

## 1. Introduction

As traditional engineering materials are quickly achieving their performance limits, there is a pressing need for next-generation materials for a wide range of applications in the biomedical, robotics, and aerospace fields. New demands include stretchable materials for soft robotics and electronics [1,2], flexible materials for electromagnetic interference shielding [3], high-density and mechanically robust energy storage devices [4,5], and biocompatible and mechanically optimized biosensors [6,7]. These examples highlight the multifunctionality requirements of next-generational materials. The critical functional focus, such as biosensing, electromagnetic interference (EMI) shielding, or damage tolerance, needs to be balanced with structural requirements of toughness and flexibility. Balancing functional needs and structural requirements necessitates discovering new materials and design traits.

Recently, several classes of two-dimensional (2D) materials (both elemental and hetero-nuclear) have been synthesized, including graphene, borophene, transition metal dichalcogenides or TMDs, and MXenes. Each of these material classes has a unique combination of mechanical, chemical, and electrical properties [8,9,10,11,12,13]. Significant effort has focused on their processing and identifying appropriate applications for them [13,14]. Among these material classes, MXenes is the most recent entry and has a general representation of $M_{n+1}X_{n}T_{x}$ (M = early transition metals, X = carbon or nitrogen, and T = functional surface terminations). MXenes can exist in several forms through element permutations and surface terminations and, thus, offer an exciting opportunity for a wide range of applications [8,14,15,16,17].

Overall, the 2D materials from the early discovery of graphene to the new entry of MXenes are attractive for multifunctional applications. They typically have a high in-plane specific area and high in-plane stiffness but are relatively weak in shear and have low out-of-plane stiffness and mechanical flexibility [18,19,20,21,22]. Their scalability beyond the lab continues to challenge real-world applications from processing to mechanical robustness aspects [19,23,24]. While there has been an immense focus on synthesizing such materials, strategies for structural stability for designs have received far less attention.

Some proposed strategies for improving the mechanical robustness of 2D structures have included using flexible substrates [20,25,26,27] and composite design [3,27,28,29,30,31,32]. Here, we focus on layered composite design for multifunctionality, wherein the target is to achieve mechanical robustness without sacrificing the primary benefit of the use of these materials. Some examples of multifunctional benefits include providing an ultrathin layer with high electrical conductivity in flexible electronics [3], providing a large surface area in gas sensing [33], and incorporating impact resistance with electromagnetic shielding [34]. Such multifunctional designs require a high concentration of 2D materials and a careful selection of design features to achieve the performance demands across applications.

Natural composites provide an excellent template of tightly balanced design traits optimized over several millennia through evolutionary forces [35,36]. Bioinspired features have guided the development of multiple engineered solutions in recent years [37,38,39]. In the context of 2D natural systems, layered architecture with carefully designed interfaces is the hallmark of many natural composites, allowing them to achieve strength and functionality far exceeding their constituents [35,36]. For example, despite being made from more than 95% mineral, nacre achieves three orders of magnitude of higher fracture resistance than its mineral phase through its unique brick-and-mortar organization and careful design of organic–mineral interfaces [40,41]. Despite having 88 vol% mineral content, the exoskeletal forewings of the diabolical ironclad beetle (*Phloeodes diabolicus*) achieve extreme toughness through a combination of design features, including laminated microstructure, spatially varying stiffness, and ellipsoidal geometry [42]. The ultrathin coating (≈70 μm) on the dactyl club of mantis shrimps has 88 vol% densely packed hydroxyapatite nanoparticles, but it achieves high toughness and impact resistance through an interspersed organic matrix within its nanoparticles [43]. The geomaterial sheet silicates montmorillonite (MMT) or nanoclay is another example of a 2D composite that has been extensively used for membrane separation and as a flame retardant due to its increased thermal stability, hydration swelling, and water dispersion properties [44,45,46,47].

Exploring the structure–function relationship of these highly mineralized thin-layered natural composites provides an excellent template for designing 2D-engineered composites. With that aim, this paper reviews the structural organization and mechanics of highly mineralized layered natural composites to identify critical design traits governing their stiffness and toughness. Following the above, we discuss the relevance of these bioinspired design features for 2D-engineered composites and some exciting challenges and opportunities for future work in 2D nanocomposite design.

## 2. Highly Mineralized 2D Natural Composites

**Figure 1:**
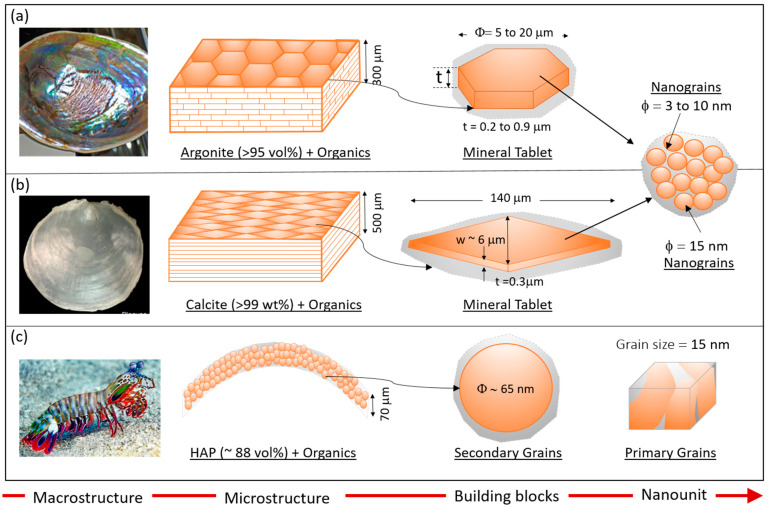
Structural organization of thin mineralized composite (**a**) nacre, (**b**) exoskeleton of windowpane oyster (WO), and (**c**) outer coating on dactyl club of mantis shrimp (DCMS) showing the mineral-organic organization at the microscale, underlying building block, and the nanounit within the building block (macrostructure courtesy: nacre [73], windowpane oyster [66], and mantis shrimp [43]).

Classic examples of 2D natural composites with high mineralization (>80 vol%) include nacreous shells, the exoskeleton of windowpane oyster (WO), and the thin outer coating on the dactyl club of mantis shrimp (DCMS). Due to their superior mechanical properties, specifically high toughness and low compressive to tensile strength ratios, the structural organization and underlying mechanisms driving their behavior have been the focus of intense investigations [35,36,41,48,49,49,50,51,52]. While the nacre and exoskeleton of WO have layered organization, the DCMS structure is nanograined and possesses overlapping features of relevance, such as high mineralization and mineral–polymer interactions, which are responsible for their superior toughness under impact loading. Furthermore, as we will discuss later, nanogranularity is an overlapping feature present not only in DCMS but also within the tablets of nacre and WO. Hence, DCMS is also included in the discussion below on natural systems of relevant layered composite design. Here, some key aspects of the structural organization are briefly summarized to identify and compare their design features for driving further discussions. Figure 1 shows the structural organization of the three natural composites.

### 2.1. Structural Organization

Nacre is a classic example of a 2D layered structure and is present in the inner surface of the exoskeletal of certain molluscan shells. It is primarily made from stiff mineralized tablets (≈95 vol% aragonite or ${CaCO}_{3}$) interspersed with soft organic polymer phases (polysaccharide and hydrated protein) in a brick-and-mortar architecture [53,54]. Figure 1a shows the hierarchical organization of nacre derived from molluscan shells. The “brick” is the aragonite present as a continuous lamellar sheet of plate-like polygonal tablets 5 to 20 μm in diameter and 0.2 to 0.9 μm thick, with an aspect ratio of 8 to 14 [41,55,56,57]. Each tablet is “glued” to the adjacent tablets via a 20 to 50 nm intra-tablet organic “mortar” layer [40,58]. The tablets are not single-crystal but instead are made from clusters of “mesocrystal” comprised of polygonal aragonite nanograins 3 to 10 nm in size, again glued using the interspersed polymer phase between the nanograins [59,60,61,62]. Multiple parallel tablet–polymer zones are arranged in a staggered brick–mortar organization to achieve a total thickness of 300 μm [40]. This tiled nacreous zone is sandwiched between mesolayers 20 μm thick, which is also made of a mineral phase ${CaCO}_{3}$ with interspersed organic phase [40,63,64]. The mesolayers are also called the growth band since they are believed to originate during the growth phase due to the variation in the feeding and temperature patterns, resulting in interruptions in the brick–mortar layering pattern.

The exoskeleton of WO (*Placuna placenta*) is another example of a highly mineralized structure (≈99 wt% calcite or ${CaCO}_{3}$), which can simultaneously achieve high optical transparency, stiffness, toughness, and high-strain impact resistance [65,66]. Like nacre, it has a lamellar organization made from mineral tablets and interspersed organics. The tablets are elongated diamond-shaped calcite tiles (length 140 μm, width 6 μm, tip angle 10°, and thickness 0.3 μm) glued together by an ultrathin (≈2 nm) organic phase (Figure 1b). The entire shell comprises 2000 such laminar layers, leading to a total thickness of approximately 500 μm. Many $\left(10^{6}\right)$ pairs of screw-like connection centers are present within each layer to provide growth pathways for biomineralization [67,68,69]. These connection centers also play a significant role in energy dissipation via interlocking of tablets and localizing damage [65], as discussed in later sections.

The outer coating of DCMS is another example of mineralized material (≈88 vol% hydroxyapatite or HAP) designed for impact resistance [43,70,71]. Its structural architecture differs from the tablet-like laminated organization of nacre and WO but shares with them the nanogranularity feature present within the tablets of layered systems and here at two different length scales (primary and secondary grains). The ultrathin (≈70 μm) outer coating is made from dense packing of crystalline HAP nanoparticles or secondary grains (≈65 nm) embedded in an organic matrix (polysaccharide and protein). The nanoparticles are not single crystals [71] but instead consist of highly aligned primary grains (≈15 nm) of preferred orientation, separated from adjacent primary grains with low-angle grain boundaries [43]. The hydrated organic matrix interpenetrates the primary and the secondary grains, similar to the organic material in inter- and intra-table regions of nacre and WO. The grain boundaries and the organic phase provide pathways for energy dissipation and crack localization, leading to extreme damage resistance under high impact [43,72], as discussed in later sections.

### 2.2. Mechanical Response

**Figure 2:**
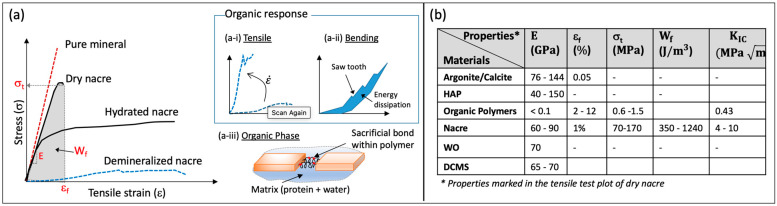
(**a**) Schematic views of stress–strain response of nacre under dry and hydrated conditions based on properties reported in the literature and (**b**) summary of typical values reported for constituent phases and corresponding natural composites (E = Elastic modulus, $\varepsilon_{f}$ = strain to failure, $\sigma_{t}$ = tensile strength, $W_{f}$ = work of fracture measured as the area under the stress–strain curve, and $K_{IC}$ = mode-I fracture toughness). The inset in (**a**) shows the response of the organic phase under tensile (**a-i**) and bending (**a-ii**) loading, with some unique characteristics revealed, including strain-rate sensitivity, saw-tooth pattern, and energy dissipation. These characteristics can be explained through the presence of a hydrated matrix comprised of organic polymers with helical polymer structures and sacrificial bonds (Figure (**a-iii**)). Data are taken from multiple sources: aragonite and calcite [53,74,75,76], HAP [77,78], organic phase [53,79,80,81], nacre [59,82,83,84,86], WO [65,66], and DCMS [70,87].

The structural organization of the nacre, the exoskeleton of WO, and the outer coating of DCMS show examples of highly mineralized composite made of nanogranulated minerals organized either in laminar tablets or in crystalline grain-like structure, interspersed with soft organic materials. The above structural organization leads to their unique mechanical response. Figure 2a shows a schematic of the typical stress–strain response of nacre and its constituents. Figure 2b shows values of some of the common mechanical properties of these natural composites and their constituents.

The minerals aragonite and calcite are brittle and orthotropic with elastic moduli between 76 and 144 GPa and a low strain to failure of 0.05% [53,74,75,76]. Similarly, HAP is brittle with a modulus of 40 to 150 GPa, the higher of the values reported for synthetic crystals [77,78]. The organic polymers (polysaccharide and hydrated protein) have a significantly lower modulus of 1 to 136 MPa depending on the strain rate, low strength of 0.6 to 1.5 MPa, and fracture toughness $K_{IC}$ of 0.43 ($MPa\sqrt{m}$) [53,79,80,81]. However, it shows significant strain to failure (2 to 12%), deformation strengthening, and viscoelasticity [79,80,81]. In contrast to their constitute phases, all-natural composites offer a remarkable combination of properties. Nacre has been the most widely studied material among the three systems. It has a tensile elastic modulus of 60 to 90 GPa comparable to its mineral phase and shows strain hardening post-yield with large strain to failure (≈1 to 8%) [41,59,82,83,84]. The tensile strength varies from 70 to 170 MPa with a work of fracture $W_{f}$ between 350 and 1240 $J/m^{3}$ [41,82,84]. Its fracture toughness $K_{IC}$ is reported between 4 and 10 ($MPa\sqrt{m}$) [84,85,86]. These properties of nacre are in stark contrast to its primary constituent, aragonite, for which the $W_{f}$ is 3000 times lower, and $\sigma_{f}$ and $K_{IC}$ are almost 20 to 30 times smaller [51,86].

Though limited experimental data are available for WO [65,66] and DCMS [70,87], they show a similar trend as nacre, unique from their primary mineral constituent. Indentation tests on freshly cleaved WO reveal a high elastic modulus of 70 GPa that is comparable to the modulus of a single calcite crystal but a substantially increased plasticity with 55% higher hardness (3.8 GPa vs. 2.5 GPa for calcite) and localized damaged response [65,66]. A theoretical calculation of interfacial energy dissipation in WO reveals interfacial fracture toughness $e_{1}$ of 100 $J/m^{2}$, two orders of magnitude higher than its constituent mineral crystal [65]. Unlike the nacre and exoskeleton of WO that fail under high strain rate impact [36], DCMS coating can resist high-velocity impact [70,87]. It has a similar modulus of 65 to 70 GPa as nacre and exoskeleton of WO [70,87]. These data show that the structure is highly optimized for extreme toughness and fracture resistance, far higher than its constituent materials.

The properties reported above have high sensitivity to hydration, as revealed through testing of nacre. Figure 2a schematically shows the typical response under dry and hydrated conditions, illustrating the hygromechanical sensitive response. Lower modulus and higher failure strain correspond to a hydrated state of testing [56,84,88]. Hydrated nacre also shows higher plasticity and viscoelasticity, observed through the hysteresis loops in tension or material pile-up in indentation [83,89,90]. The property modification of nacre in the presence of water can be attributed to multiple factors, such as the plasticizing effect of bulk water presence in inter and intra-tablet mineral gaps and pores, a reduction of surface energies through chemical absorption of water on mineral surfaces, and the stabilization via hydrogen bonding of the organic phase [84,91,92]. These factors enable a gradual stress transition from the organic phase to the tablet interfaces during sliding, resulting in higher toughness [92]. Consequently, the properties of nacre, specifically its toughness and high strain rate sensitivity, are highly influenced by its organic phase. The insets (a-i) and (a-ii) of Figure 2a show the typical response of the organic phase. The tensile curve shows high strain-rate sensitivity. The bending curve shows variable stiffness under loading with saw-tooth-like patterns and energy dissipation upon unloading. Many of the features of the response of hydrated nacre and other natural composites can be attributed to the mechanical influence of the hydrated matrix explained above. However, other features, such as its helical structure and sacrificial bonds, also play an essential role and are discussed later.

## 3. Design Features of Mineralized Composites for Stiffness and Toughness

Several design features lead to the extraordinary mechanical response of these highly mineralized composites, which can be assigned to three broad categories, namely (a) nanogranular organization, (b) mechanical interlocking of tablet interfaces, and (c) interphase zone of organic materials. These key design traits are discussed next, along with other mechanisms, such as deformation twinning and aspect ratios of tablets, which influence the mechanical response.

### 3.1. Nanograin Architecture Drives Flaw Resistance

The nanogranular arrangement within the tablets is a crucial design feature of biomineralized composites. In nacre, nanograins 3 to 10 nm in size are bonded together to form a polygonal tablet [59,62]. The size of the granules can vary within a tablet, thereby controlling the density distribution of organic interphases and, consequently, inhomogeneity in elastic modulus, leading to improved fracture response [62,93,94]. In the exoskeleton of WO, the elongated diamond-shaped calcite tiles are also comprised of nanograins ≈ 50 nm in size [66]. The coating of DCMS has a more apparent nanogranular organization than that which occurs within the tablets of nacre and WO. The nanogranules are present as secondary grains ≈ 60 nm in size and as aligned primary grains ≈ 15 nm in size, which are organized within the secondary grains.

The nanoscale organization of natural materials has been explained through crack tolerance design [95]. Using Griffith criteria and an idealized laminar architecture consisting of staggered mineral tablets in a protein matrix, equations for the fracture response of the mineral tablet were derived, as given by Equations (1) and (2). The fracture strength of the tablet at failure ($\sigma_{m}^{f}$) is given by Equation (1), and the critical thickness ($h^{*}$) for its fracture strength to be identical to the strength of the perfect crystal is given by Equation (2). The value of $h^{*}$ is calculated as 30 nm for values $\gamma =1\;{J/m}^{2}$, $E_{m}=100\;GPa$, and assuming $\sigma_{th}=E_{m}/30$ [95].

(1) $$\sigma_{m}^{f}=\alpha E_{m}\sqrt{\frac{\gamma }{E_{m}h}}$$

(2) $$h^{*}\approx \alpha^{2}\frac{\gamma E_{m}}{\sigma_{th}^{2}}$$

where h = thickness of mineral platelet, $h^{*}$ = critical thickness of mineral platelet, $\sigma_{m}^{f}$ = fracture strength of mineral platelet, $\sigma_{th}$ = theoretical strength of perfect mineral crystal, γ = surface energy of mineral (${J/m}^{2}$), $E_{m}$ = elastic modulus of mineral, and $\alpha \approx \sqrt{\pi }$ for half-cracked platelet.

While the above method is a simplified analysis for an idealized tablet without accounting for geometrical constraints or the strength contribution of the organic phase, it explains the nanogranular organization as a means for improving crack tolerance. These relationships also provide a method for comparing crack tolerance responses across natural composites. For example, the nanograins in nacre (3 to 10 nm) are below the h* values, which indicates its tablet design emphasizes toughness over maximizing strength. The larger granules within the tablet of WO (≈50 nm) compared to a finer organization of nacre (3 to 10 nm) and DCMS coating (15 nm) predicts that tablets of WO are the least resistant to tablet cracking of these three material systems.

### 3.2. Mechanical Interface Interlocking Increases Strength and Toughness

**Figure 3:**
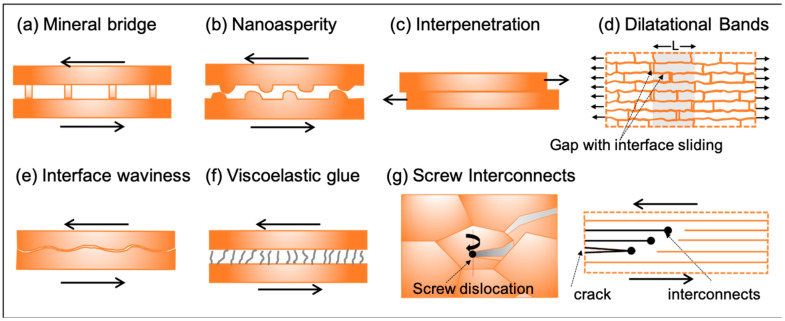
Common mechanical interlocking mechanisms for tablet interfaces include (**a**) mineral bridging, (**b**) nanoasperity, (**c**) tablet interpenetration, (**d**) dilatational bands, (**e**) tablet waviness, (**f**) viscoelastic glue, and (**g**) screw interconnects. Arrows indicates primary force direction relevant to activate the mechanism shown.

The mechanical interlocking of tablets may occur by several different mechanisms, as depicted in Figure 3. These mechanisms include mineral bridges, nanoasperity, tablet interpenetration, dilatational bands, tablet waviness, tablet curvature, viscoelastic glue, and screw-like microscopic interconnects. These interlocking mechanisms improve structural integrity and modify the composite mechanical response and are reviewed in multiple sources [51,54,96,97,98].

Mineral bridges (Figure 3a) are believed to be sites for mineralization during the growth stage of tablets [80,99] and were among the early mechanisms identified for stiffening in nacre-like materials [48,52,100]. Later experiments showed mineral bridge concentration was present only in the middle regions of tablets, limiting their role to adjacent vertical stacks of tablets rather than the whole nacre level response [55]. Other research instead identified alternative mechanisms such as nanoasperity [41,48] and tablet interpenetration for interlocking [101,102] (Figure 3b,c). Nanoasperity increases shear resistance by increasing contact sites between adjacent tablets. It improves fracture toughness by providing additional surfaces for energy dissipation [41,48] but only under small sliding to about 15 to 20 nm [48,96]. The steady-state sliding post-hardening can also be explained by the tablet sliding over the nanoasperities [41,48]. The sizeable nonlinearity and strain hardening observed in nacre under tension and increased shear stiffness under sliding can also be attributed to the formation and interconnection of multiple dilatational bands [41] (Figure 3d). Mineral bridging, nanoasperities, and tablet interpenetration sites can contribute to the increase in the pull-out force of the structure under sliding [51,52,100,101,102] but cannot explain strain hardening, which was attributed to the presence of tablet waviness. Tablets surfaces have waviness (Figure 3e) up to 200 nm in amplitude [56]. This waviness further improves physical interlocking, localizes damage, and increases surface areas for energy dissipation, resulting in strain hardening and increased toughness [56,57,96]. Structural waviness for interlocking has also been observed in various other shell structures [97,103]. The polymer present in the interface and interphase regions acts as a “viscoelastic glue” by adhering to interconnecting tablets [59,63,82,104] (Figure 3f). The polymer phase is also responsible for the enhancement of a range of composite properties, which are discussed comprehensively later.

Screw dislocation interconnects that provide biomineralization pathways during growth are another mechanism identified for tablet interlocking in several laminated structures, including nacre and WO [65,65,67,68,69] (Figure 3g). These interconnects are believed to interlock tablets and offer additional surfaces for energy dissipation, thus localizing damage and improving strength and toughness [65,66,69]. A theoretical analysis of the effect on fracture energy dissipation in the presence of a pair of screw dislocations was performed for the exoskeletal of WO [44] and is given by Equation (3). In simple laminates, only $e_{int}$ mode of energy dissipation will exist, which is the energy dissipation along adjacent mineral interfaces. For WO laminated structure with screw dislocations, additional energy dissipation occurs due to the presence of an inelastic deformation zone ahead of the crack tip ($=\frac{t\lambda_{inel}}{\lambda }e_{inel})$, the formation of vertical surfaces along fracture lines ($=\frac{t}{\lambda }e_{cal}$), and the opening of surfaces along fracture lines ($=\frac{\lambda_{inel}}{\lambda }e_{inl}$) as represented in Equation (3). The above theoretical analysis predicts the energy dissipation in the exoskeleton of WO having larger tablet surfaces as two orders of magnitude higher than simple mineral laminated structures. In contrast, energy dissipation from screw dislocations will be nominal in smaller tablet sizes, such as nacre [65].

(3) $$e_{1}=e_{int}+\frac{t\lambda_{inel}}{\lambda }e_{inel}+\frac{t}{\lambda }e_{cal}+\frac{\lambda_{inel}}{\lambda }e_{int}$$

where $e_{int}$ = energy dissipation density of mineral layer (${J/m}^{2}$), $e_{inel}$ = energy dissipation density of mineral layer (${J/m}^{3}$), $e_{cal}$ = surface energy along the cleavage plane of calcite (${J/m}^{2}$), t is the thickness of mineral layers (m), λ = spacing between two adjacent fracture lines, and $\lambda_{inel}$ = inelastic deformation zone (m).

### 3.3. Interpenetrated Organic Phases Provide Structural Integrity, Plasticity, and Toughness

The organic phase is mechanically weak compared to the mineral phase but shows viscoelasticity, deformation strengthening, and significant strain to failure [79,80,81] (Figure 3). Given its low strength and small volume contribution (<5 vol%), many of the earlier studies focused primarily on the mineral phase, limiting the role of polymer interphases only as “viscoelastic glue” for structural integrity and shear strength modifications [59,63,82,104]. Studies have now revealed a broad role of organic phases in natural composites, from crystal formation to hygromechanical property modification [40,80,88,90,98,104,105,106,107,108]. For example, the brittle response of nacre in the absence of hydration indicates that most of its shear load is carried by the hydrated organic phase [82]. The presence of stiff and coiled component chitin and folded structure of protein lustrin A, along with the presence of nanopores 5 to 50 nm in diameter within the organic phase, can explain the adhesion, stiffness, and large deformation of nacre [58,80,90,109].

**Figure 4:**
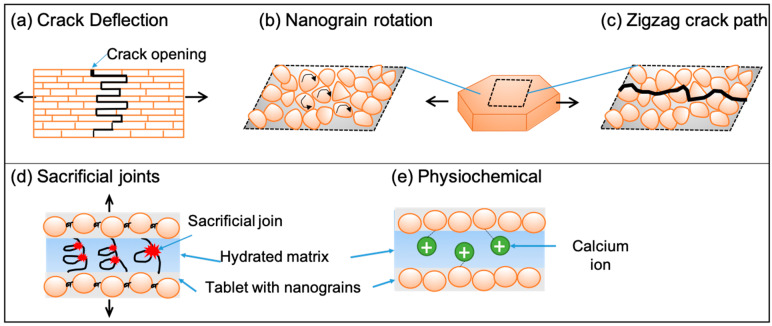
Common mechanisms highlighting the role of organic materials beyond acting as viscoelastic glue include (**a**) pathway for viscoelastic energy dissipation, (**b**) nanograin rotation, (**c**) zigzag path for crack, (**d**) sacrificial joints, and (**e**) physiochemical interactions of polysaccharide and proteins structure in the presence of water. Arrows indicates primary force direction relevant to activate the mechanism shown.

Figure 4 shows some of these mechanisms, highlighting the important and broader role of the organic layer. The intra-tablet distribution of the organic layer increases energy dissipation pathways by crack deflection [51,110,111] (Figure 4a), thus affecting nacre toughness. Nanogranularity and interspersed organic matrix within the tablet together allow for nanograin rotation under load, increasing plasticity deformation and energy dissipation paths for toughening of individual tablets [49,112] (Figure 4b). Nanogranularity also increases energy dissipation and flow tolerance within tablets by creating a zigzag path for crack motion [113] (Figure 4c). Other mechanisms contributing to toughening and large strains of the tablets include varying density distribution of the interspersed polymer for elastic modulus gradient [62,93,94] and anisotropic lattice distortion [114,115]. The “saw-tooth” pattern in pull tests reveals the presence of sacrificial bonds within the organic layer (Figure 4d), which results in significant deformation and stiffening [88,90,108]. These bonds are reversible and, hence, also provide self-healing properties to some natural composites [108]. Finally, various physiochemical interactions in the presence of water are also said to be responsible for adhesion and hygromechanical sensitivity [116] (Figure 4e). Increasing hydration results in polymer softening and provides increased plasticity and viscoelasticity effects [117,118,119,120]. Similarly, a significant loss in toughness and tribological wear is observed under lower levels of hydration [116,121]. Beyond the apparent impact of polymer plasticization, the presence of physicochemical interactions influences the hygromechanical sensitivity in natural composites.

### 3.4. Deformation Twinning Can Be Prominent for Damage Localization

Though less common than dislocation and grain boundaries, twinning is a defect in crystalline structures like metals, allowing local plastic deformation [122,123]. Recently, nanoscale deformation twinning has been identified as a damage localization mechanism in some natural composites [103,124,125,126,127]. For example, nanoscale twinning 2 to 20 nm thick was found within aragonite lamella of conch shells (*Strombus gigas*) and was recognized as an essential mechanism for toughening and localization of damage [103]. A deformation twin ≈ 50 nm thick was formed around the damage zone of indentation in the exoskeleton of WO at the earliest deformation stage, causing damage localization at the early stages, followed by other mechanisms such as interface opening and micro/nano cracking for toughening [127]. Nanoscale twinning is also present in nacre, though it has a much lower influence on mechanical response [127].

### 3.5. The Tablet Aspect Ratio Influences the Transition from a Tougher to a Stiffer Response

The tablet sizes and geometry vary among the different composites. Nacre tablets are polygonal 5 to 20 μm in diameter with an aspect ratio (AR) from 20 to 100. Those of the exoskeleton of WO are diamond-shaped with a maximum length of 140 μm and AR of approximately 500, much higher than nacre. Finally, in the extreme case of DCMS, the “tablets” reduce to nanoscale size. The aspect ratio is an essential feature of natural composites, determining the relative contribution of different deformation mechanisms [57,95,128,129]. For example, larger aspect ratios and staggered alignment can increase strength at the cost of ductility [129,130]. Screw-like interconnects can significantly increase strength for larger tablets like WO but have a negligible effect on smaller tablets like nacre [65]. In contrast, larger tablets are more susceptible to pull-out damage and fracture developing within their surface. These, in turn, indicate that an optimal tablet size or geometrical features should be guided by the functional demand of the composites and can be predicted by theoretical analysis of idealized composites [95,118,131,132].

Based on the laminar model architecture consisting of staggered mineral platelets in a protein matrix discussed earlier (Equations (1) and (2)) and assuming simple force distribution between mineral and protein, a critical value of aspect ratio (${AR}^{*}$) was derived for optimum strength and toughness [95]. The value is given by Equation (4) under the simultaneous failure of the mineral and polymer phase, which shows that the optimum aspect ratio is inversely proportional to $\sqrt{h}$. It implied that thinner tablets need higher aspect ratios for a similar stiffening effect. An optimum AR of approximately 25 was obtained for typical values for mineral and organic constituents ($\sigma_{m}^{f}$ = 1 GPa and $\tau_{o}^{f}$ = 40 MPa) [65]. Values higher than the optimum values will have higher stiffness at the cost of their toughness and vice versa. A similar prediction was made for general 2D composites, where ductile matrix deformation dominates at lower than a critical value of AR, while platelet fracture mode results at larger values. The exact value of AR will depend on the tensile strength of reinforcement [118].

(4) $${AR}^{*}=\frac{\sigma_{m}^{f}}{\tau_{o}^{f}}=\frac{1}{\tau_{o}^{f}}\sqrt{\frac{\pi E_{m}\gamma }{h}}$$

where $\sigma_{m}^{f}$ = failure stress of the mineral phase, $\tau_{o}^{f}$ = shear failure stress in the organic phase, and $E_{m}$ and γ are as defined in Equation (1).

Based on the above theoretical analysis, the exoskeleton of WO with higher AR should address higher stiffness applications. At the same time, a smaller AR of nacre or bone should be designed for greater toughness. This prediction matches the understanding of their comparative performance and application. As a further confirmation, the toughness of nacre is three orders of magnitude larger than its mineral constituent, and that of the exoskeleton of WO is only two orders of magnitude larger. Another tablet size-related design feature is the thickness of the organic interphase between tablets. However, the thickness of the organic phase cannot be treated independently; it depends on the volume contribution of the organic phase and the size of the tablets. A smaller thickness (≈2 nm) is present in the exoskeleton of WO, which has a relatively small volume contribution of the organic phase (≈1%) and larger tablets. In comparison, a larger thickness (≈20 nm) is present in nacre, which has a more considerable volume contribution of the organic phase (≈5%) and smaller tablets. The discussion on the impact of various design features of mineralized 2D composites on the mechanical response is summarized in Table 1.

## 4. Application of Bioinspired Design Rules for Engineered 2D Composites

The review above and Table 1 summarize the critical design features and structural organization of mineralized 2D materials to address mechanical demands for a tough armor-like response. Many of the same design features are also optimized for other functional demands of these structures, such as surface smoothness, iridescence, and optical transparency for parasitic defense for most shells [66,133,134,135] and high strain impact resistance for hammer-like function for DCMS [43,70]. In general, the bioinspired design space is exhaustive [36,136,137,138,139,140], with many nature-mimetic designs engineered for a range of functional demands from damage tolerance [140,141,142,143,144,145,146,147], tunable transparency [118,133,148], improved aerodynamics [138,149,150,151,152], thermal management [118,149,153,154,155], and as a gas barrier [120].

**Figure 5:**
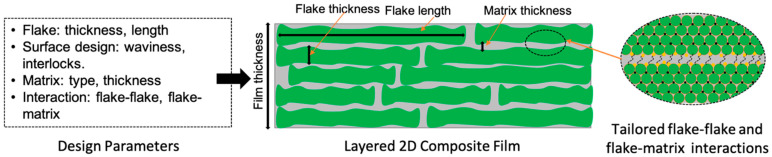
List and schematic of available parameters for layered 2D material–polymer composite design.

Figure 5 shows a typical layered film and associated design parameters that can be influenced by bioinspired parameters, including flake thickness, flake length, choice of a polymer matrix, matrix thickness, flake–flake and flake–matrix interactions, and surface design. The section below discusses these features in the context of bioinspired design and related challenges, along with illustrating examples of 2D material-based composites with those design features.

Flake Geometry: To mimic tablet influence, balancing strength with toughness requires control of aspect ratio. Tablets in natural composites are highly organized and uniform. Tablets of larger AR > 100 provide stiffness, but such tablets negatively impact the toughness. Flake sizes in 2D materials are highly dependent on process parameters and can vary significantly for the same technique. For example, MXene/TAEA sheets processed by layer-by-layer self-assembly resulted in AR 80 to 500 with improved electrochemical performance [156]. In another study, acid etching resulted in MXene flakes with a much higher AR of 6000 [157]. In a study on graphene, graphene flake was prepared by a non-dispersive method in a slurry form, which can be used for creating high-concentration graphene composite using 3D printing [158]. Cellulose nanocrystals (CNC) have been used to stabilize graphene flakes, resulting in a layered graphene/CNC composite [159]. Another graphene composite production resulted in conductive paper from multilayered graphene flakes of 0.3 to 0.4 μm in polyvinyl alcohol (PVA) [160]. Multiple other layered nanocomposites have been produced [161,162,163,164], the discussion of which is beyond the scope of the article. However, across all these different processes and 2D material types, controlling the processing parameters for targeted flake geometry remains immature. Understanding the impact of tablet size and aspect ratios is thus helpful in directing the research on MXene and graphene formation toward targeted and uniform-sized flake processing.

Interface interlocks: Another bioinspired feature of significant influence is interface interlock-induced strength enhancement. Many earlier mechanical strategies for creating tablet connections in other materials have shown severe limitations. Methods like tablet stitching and creating nanotube forests improve out-of-plane stiffness and pull-out forces but lead to defects and damage within the original laminates due to the formation of stress concentration sites [165,166,167,168]. Microcapsules between layers can also lead to surface roughness for shear properties via interface interlocks [169]. Other interface mechanisms, such as stitching via the formation of screw-like dislocation centers inspired by the exoskeleton of WO, roughened interfaces, surface waviness, and other alternatives for interface design, can impact response and could be explored via processing.

Polymer interphases: Tablet interlock via polymer interphases, similar to the role of organic polymers in natural composites, is another bioinspired strategy that could be employed for shear strengthening and improving toughness. The organic polymeric material is crucial for the structural integrity, plasticity, and shear rigidity of the mineralized 2D natural composites through multiple mechanisms, as highlighted in Figure 4. Molecular bond interconnections of 2D sheets with interphase polymer in a layer-by-layer deposition have been utilized over the years to give a nacre-like interphase bonding [170,171,172,173,174,175]. Increased stretchability in graphene-based composite was obtained by ionic bonding by adding ${Ca}^{2+}$ ions [176,177]. Hydrogen bonding between the slightly hydrophobic amine-terminated silane ($SiH_{4}$) with oxygen atoms of chitosan increased the polymer’s cohesion to alumina tablet for properties comparable to nacre [178]. A similar moisture-based actuation with high electrical conductivity was also achieved in a homogenous MXene sheet utilizing the hydrophilic nature of its surface termination layers [179].

Hence, multiple polymer interphase strengthening strategies have been well developed over the years for many different types of nanocomposites. Furthermore, multiple synthetic and natural polymers are available to mirror the mechanical properties of the organic phase. Some synthetic polymers used in the nacre-mimetic design include acrylic foam tapes [139], PVA [180,181], poly-diallyl dimethylammonium chloride (PDDA) [182], poly-methyl methacrylate (PMMA) [181,183], polyacrylic acid (PAA) [184], and polyelectrolyte multilayer (PEM) [185]. Natural polymers used include chitosan [178], silk fibroin [146,176,177,186,187], alginate, and CNF [180,188]. While many of these polymers can capture the mechanical stiffness and viscoelasticity of the organic phase, a comprehensive public database of the electromechanical and thermal properties of these readily available polymers could help identify the most suitable choice for a particular set of 2D material applications.

## 5. Discussion and Future Outlook

The recent development of MXenes and other 2D materials provides many opportunities and challenges to move the field of multifunctional composites forward. The paper focused on the highly mineralized structure of 2D natural composites as a pathway forward for the bioinspired design of engineered 2D composites.

In this paper, we present unique properties of the natural system different from its dominant brittle mineral phase, emphasizing the role of their structural organization and interspersed soft organic phase. We then provide a mechanistic basis for the superior properties of the layered natural system and identify several underlying design rules that determine the balance between toughness and stiffness (Table 1). Some key design traits identified include (a) nanogranular organization, (b) mechanical interlocking of tablet interfaces, (c) organic interphase zone, (d) tablet aspect ratio, and (e) hydration. For example, a higher aspect ratio of the layered tablet increases the stiffness and negatively impacts the ductility and fracture toughness.

**Figure 6:**
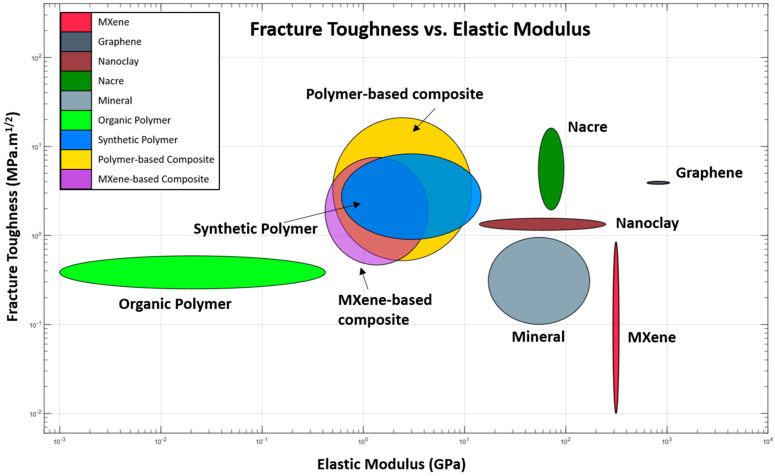
Ashby-style property space of 2D natural and engineered systems. Polymer-based composites are primarily polymers with <5% stiffening phase (graphene, MXene, nanoclay). MXene-based composites have >50% MXene. The ellipses are plotted using mean and standard deviations of maximum and minimum values of the properties reported [30,36,41,53,59,65,66,70,74,75,76,77,78,79,80,81,82,84,85,86,87,96,157,157,175,190,191,192,193,194,195,196,197,198,199,200,201].

Overall, the brick–mortar organization of nacre and WO provides one of the most straightforward systems for replication compared to other more complex hierarchical organizations, such as bone and plants [189]. However, translating bioinspired design features to engineered systems offers many unique challenges and remains to be achieved. For example, Figure 6 shows Ashby-style property space of strength and toughness for a natural system and engineered 2D materials (MXene, graphene, and nanoclay) and their composites. The figure highlights the large gap in the mechanical properties of the engineered 2D systems compared to their bioinspired counterparts, which further emphasizes the need for research and growth in that area.

Some of these challenges in the context of structural design and processing are discussed in Section 4. These include (a) control over the lateral size of flakes thickness, (b) improved understanding of the relative impact of mechanical interlocking mechanisms such as screw connection vs. wavy interfaces, (c) challenges in processing controlled mechanical interfaces either mechanically or through surface functionalization, and (d) the need for a public database of the electromechanical and thermal properties of readily available synthetic and natural polymers. Identifying and overcoming these challenges can enable achieving the theoretical limits of mechanical response in engineered 2D systems and provide multifunctional capabilities far above those of more traditional materials. Below are some perspectives on the future outlook for growth in the design and manufacturing of bioinspired engineered 2D systems.

Atomic-Scale and Integration with Higher-Scale Simulation: With the rapid advancement in processing, a large family of 2D materials has been synthesized for many different applications [14,202,203,204,205,206]. Given the wide choice of homo- and hetero-nuclear 2D materials, different types of surface termination layers, and novel synthesis pathways, there is a tremendous opportunity for systematic integration of atomic-level simulation with experiments to guide materials and process choices and provide an integrated development, testing, and validation platform. Due to recent successes in the experimental realization of various 2D materials, such simulations are currently very limited for most 2D materials but show their predictive capability. Considering MXenes as an example, recent molecular dynamics (MD) simulations on titanium-carbide (TiC)-based MXene predicted its tensile elastic modulus between 500 and 600 GPa, the nonlinear transition at <1% strains, high strain to failure (6 to 9%), and temperature and strain-rate sensitivity [157,192,207,208]. The binding energy was obtained between 0.8 and 2 × $10^{-18}\;J$ [209]. Indentation experiments on ${Ti}_{3}C_{2}T_{x}$ revealed a lower elastic modulus of 300 GPa [157,192] than those predicted from these simulations. Such atomistic simulation studies need to be extended to create a comprehensive database relating MXene compositions and surface terminations with its electromechanical and electrochemical properties and explain the discrepancies between atomistic predictions from in situ values. This database will enable improved modeling fidelity and create a validated platform to guide developments and reduce the effect of experimental unknowns (surface roughness, interface/interphase chemistry, lateral sizes, thickness distributions, etc.) [210,211].

Furthermore, integrating atomic models with higher-order analysis is essential for modeling larger realistic geometries and processing. Specifically, MD analysis can be combined with multiscale continuum mechanics-based finite element analysis (FEA) to address the size limitations of atomistic simulations and provide realistic properties. For example, higher-order continuum analysis not only requires the stress–strain response of 2D sheets but also needs a measure of interface strength (surface interaction energies, frictional/shear response) to model slippage and adhesion between flake-to-flake and flake-to-polymer surfaces. Such properties are complicated and time-consuming to measure through experiments. Hence, integrated MD/FEA simulation will serve the dual purpose of using realistic interactions and material properties along with bridging experimental outcomes. Without such material-specific information, FEA application for 2D composite design will be severely limited to elastic response and rigid contact interactions such as those used in recent works [212,213,214].

**Figure 7:**
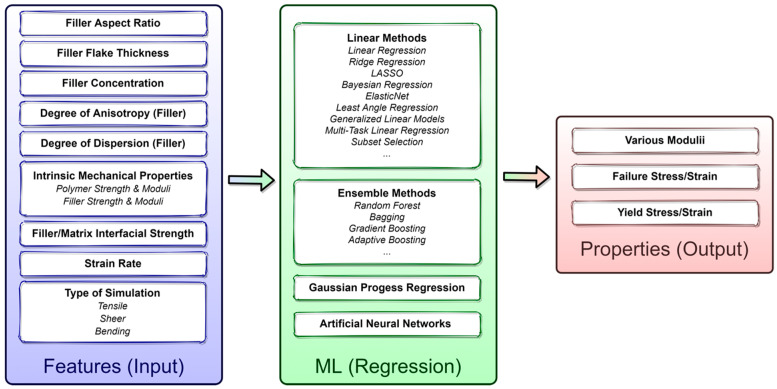
Integrated platform for accelerated development of multifunctional 2D-engineered composite.

Integration with Machine Learning: Machine learning (ML) is rapidly becoming an impressive tool in the discovery and design of new materials, including composites [215,216,217,218,219,220]. Integration of ML with MD or FEA simulation is also emerging [221,222,223,224]. Learning from the recent literature on composites, ML could facilitate the accelerated development of 2D materials-based polymer composites for targeted structural applications. For example, an opportunity in such composite space is shown in Figure 7, where several regression-based ML methods, including artificial neural networks (ANNs), as well as active-learning methods, such as Gaussian process regression (GPR) (especially if the training dataset is small), could be employed to understand better the correlation between input composite features and elastic/failure structural properties (strength, toughness, moduli). Here, input features could be described via a set of descriptors using intrinsic properties of 2D materials (i.e., flakes), polymer and their interfaces (such as the mechanical response of pristine systems, interface strength between the polymer and the 2D flakes), as well as morphological properties (2D flakes concentration, some measure of dispersion quality, orientational anisotropy, etc.). The trained models may assist in exploring and exploiting multi-dimensional parameter space much more efficiently and help identify optimal input features for a given structural requirement much faster. Overall, integrating multiscale modeling (MD, FEA) and experimental data of synthesized 2D materials provides an exciting opportunity for growth into physics-informed ML models.

Theoretical Modeling: A simpler theoretical analysis using idealized geometry has driven an understanding of design features for natural composites [57,95,128,129]. For example, the presence of screw dislocation increases surfaces for energy dissipation [65], and increasing tablet aspect ratios can predict the transition of the composite from low tolerance to a stiffer response [57,95,128,129]. However, these models do not incorporate the role of interfaces. Modifications to the existing theoretical models of natural composites or new theory and model development to include polymer/mineral interface or interactions could provide a more accurate prediction of their role in the mechanical response and correspondingly guide future developments toward the bioinspired design of engineered 2D systems.

Surface Chemistry Control: Beyond simulation and theoretical modeling, the development of new surface chemistries for precise control of molecular energy dissipation and tunable and sacrificial bond interactions are needed to control interface interactions. For example, incorporating small molecules during the exfoliation process of 2D materials, altering their layering, or introducing different surface chemistries can impact interfacial behavior, including adhesion and separation energies. Another aspect of surface chemistry control comes from environmental stability, especially in applications where surface oxidation may adversely affect multifunctional performance. Humidity can lead to oxidation of 2D surfaces and degrade the mechanical properties of natural polymers [117,118,119,120]. Finding pathways for stability against humidity damage becomes critical when using natural polymers such as chitin and CNF. Stabilization methods could involve new interaction development via chemical cross-linking, switching mechanisms, or thermal curing [118,225,226,227,228,229].

## 6. Conclusions

The emergence of 2D materials with exceptional property combinations has led to the rapid expansion in the use of 2D materials in various applications, from electronics to biomedical sensors, exhibiting their immense potential as versatile materials for future society. Despite tremendous interest in these materials systems, a comprehensive understanding of the underlying mechanical performance for multifunctional composite design using these 2D materials has been missing. Here, we have taken a pre-design approach, guided by natural design principles of similarly structured highly mineralized 2D natural materials, to address the challenges of 2D-based hierarchical composites for the competing requirements for strength, toughness, and surface interactions across various applications. Some key outcomes and future opportunities examined in this study are listed below.

- We have elucidated fundamental design principles drawn from highly mineralized 2D natural composites such as nacre and windowpane oyster exoskeletons, offering valuable insights for engineering superior 2D-based systems. The key design features include the nanograin tablet architecture with an increased aspect ratio critical to impacting strength at the cost of toughness. Other features include the presence of an organic interphase zone and the tablet interface interactions among themselves and with the organic phase;
- While highlighting the large gap in mechanical properties of the engineered 2D systems from their bioinspired counterparts, we have discussed the challenges of translating the bioinspired design features to engineered systems and highlighted the opportunities for research and growth. Specifically, integrating atomic models with higher-order continuum analysis is emphasized for modeling realistic geometries and interactions to guide processing;
- We have also highlighted the need for precise control of surface chemistries via processing techniques such as layer-by-layer organization with the incorporation of specialized organic interphases between layers for tunable designs. Such surface chemistries must be evaluated for their environmental stability, especially when natural polymers are integrated into the design;
- Finally, we have emphasized the need for a physics-derived validated machine learning model integrated with atomistic and continuum mechanics outcome to efficiently exploit the multi-dimensional parameter space and accelerate the design and development of 2D-based composites for real-world applications.

Overall, unlocking the full potential for MXene and other 2D material systems at an industrial scale requires careful consideration of the opportunities and challenges ahead, as identified in the paper. The future also points to an integrated role of synthesis with multiscale modeling and physics-derived machine learning approaches to pave the way forward for enabling its rapid growth and widespread utilization of these remarkable materials in practical, real-world applications.

## Figures and Tables

**Table 1:** Summary of design rules of natural composites and their impact on common mechanical properties. The arrows can be read as follows for a representative feature: as dimensions of nanograin increase, it leads to a lowering in ductility, work of fracture, and fracture toughness.

	Properties *	Elastic Modulus (E)	Fracture Strain (ε_f_)	Tensile st. (σ_f_)	Work of Fracture (W_f_)	Fracture Toughness ** (K_IC_)
Feature						
Nanograin ^+^	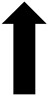		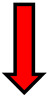	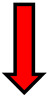	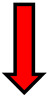	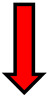
Interface interlock (mechanical)	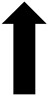	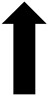	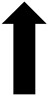	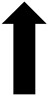	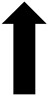	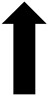
Organic lnterphases	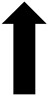	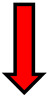	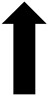	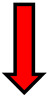	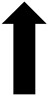	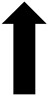
Table size/aspect ratio ^++^	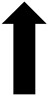	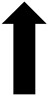	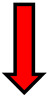	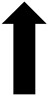	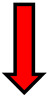	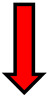
Hydration	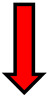	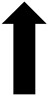	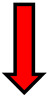	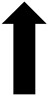	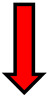	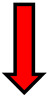

^+^ Higher than optimum depth (h*) for a given material combination; ^++^ Higher than optimum size (AP*) for a given material combination; * Hydration effect is illustrated as change in response from a fully dry state to its fully hydrated state (example:change in stress-strain of dry vs. hydrated nacre of Figure 2; ** Fracture toughness or critical stress intensity factor.

## Data Availability

Publicly available datasets were analyzed in this study. This data can be found in the references cited at relevant places.

## Abbreviations

2D	Two-dimensional
ANN	Artificial neural networks
CNF	Cellulose nanofibers
DCMS	Dactyl club of mantis shrimp
DFT	Density functional theory
EMI	Electromagnetic interference
FEA	Finite element analysis
GPR	Gaussian process regression
HAP	Hydroxyapatite
MD	Molecular dynamics
MMT	Montmorillonite
PAA	Polyacrylic acid
PDDA	Poly-diallyl dimethylammonium chloride
PEDOT:PSS	Poly(3,4-ethylenedioxythiophene)–poly(styrenesulfonate)
PEM	Polyelectrolyte multilayer
PMMA	Poly-methyl methacrylate
PVA	Polyvinyl alcohol
SE	Shielding efficiency
TAEA	Tris(2-aminoethyl) amine
TMD	Transition metal dichalcogenides
WO	Windowpane oyster
